# Self-compassion and positive body image: a role for social comparison?

**DOI:** 10.1186/2050-2974-1-S1-O54

**Published:** 2013-11-14

**Authors:** Rachel Andrew, Marika Tiggemann, Levina Clark

**Affiliations:** 1 School of Psychology, Flinders University, Australia

## 

There is continued interest in mindfulness-based therapies for eating disorders, with self-compassion identified as a key psychological construct. Self-compassion has been shown to be associated with positive body image. The aim of the present study was to examine the role of social comparison as a possible mediator of the relationship between self-compassion and both positive body image and body dissatisfaction. Participants were 266 female university students aged 18 to 29 years who completed an online survey containing measures of positive body image, body dissatisfaction, self-compassion and social comparison. Self-compassion was found to be positively related to positive body image and negatively related to body dissatisfaction. Self-compassion was found to be negatively related to social comparison. Importantly, regression analyses showed that, as predicted, social comparison at least partially mediated the relationships between self-compassion and positive body image and body dissatisfaction. The findings add to our theoretical understanding of the precursors of positive body image and provide preliminary evidence for specifically targeting self-compassion and social comparison within prevention programs and mindfulness-based therapies for eating disorders.

This abstract was presented in the **Body Image** stream of the 2013 ANZAED Conference.

